# Blood group genotyping in alloimmunized multi‐transfused thalassemia patients from Iran

**DOI:** 10.1002/mgg3.1701

**Published:** 2021-05-08

**Authors:** Reyhaneh Sarihi, Arezoo Oodi, Raziyeh Dadkhah Tehrani, Seyedeh Farzaneh Jalali, Fahimeh Mardani, Azita Azarkeivan, Samira Gudarzi, Naser Amirizadeh

**Affiliations:** ^1^ Blood transfusion Research Center High Institute for Research and Education in Transfusion Medicine Tehran Iran

**Keywords:** alloimmunization, blood groups, genotype, thalassemia

## Abstract

**Objectives:**

Serological methods may not be reliable for RBC antigen typing, especially in multi‐transfused patients. The blood group systems provoking the most severe transfusion reactions are mainly Rh, Kell, Kidd, and Duffy. We intended to determine the genotype of these blood group system antigens among Iranian alloimmunized thalassemia patients using molecular methods and compare the results with serological phenotyping.

**Methods:**

Two hundred patients participated in this study. Blood group phenotype and genotype were determined using the serological method and PCR‐SSP, respectively. The genotypes of patients with incompatibility between phenotype and genotype were re‐evaluated by RFLP‐PCR and confirmed by DNA sequencing.

**Results:**

Discrepancies between phenotype and genotype results were found in 132 alleles and 83 (41.5%) patients; however, there was complete accordance between the three genotyping methods. Most discrepancies were detected in Rh and Duffy systems with 47 and 45 cases, respectively, and the main discrepancy was in the FY*B/FY*B allele when serologically showed Fy(a+b+). All 39 undetermined phenotypes, due to mixed‐field reactions, were resolved by molecular genotyping.

**Conclusion:**

Molecular genotyping is more reliable compared with the serological method, especially in multi‐transfused patients. Therefore, the addition of blood group genotyping to serological assays can lead to an antigen‐matched transfusion in these patients.

## Graphical abstract

Molecular genotyping is more reliable compared with the serological method, especially in multi‐transfused patients. Therefore, the addition of blood group genotyping to serological assays can lead to a better‐matched transfusion in these patients.

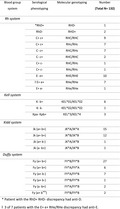

## 1 INTRODUCTION

Alloimmunization against red blood cell (RBC) antigens is the main complication associated with RBC transfusion (Kutner et al., 2014; Matteocci & Pierelli, 2014). It can cause significant health problems in chronically transfused patients, such as patients with thalassemia (Chou et al., 2012). Alloantibodies shorten in vivo survival of transfused cells, increase the need for transfusion, and restrict the number of compatible units (Chou et al., 2012; Thompson et al., 2011). Alloimmunization also triggers additional alloantibodies and autoantibody formation and can increasingly complicate further transfusion in these patients (Matteocci & Pierelli, 2014; Yazdanbakhsh et al., 2012). Accordingly, accurate RBC antigen profiling is important in thalassemia patients to reduce the risk of exposure to foreign RBC antigens and decrease the occurrence of alloimmunization (Osman et al., 2017). Serological phenotyping based on hemagglutination is a conventional method used to determine blood group antigens (Monteiro et al., 2011). However, this method has many limitations, especially in patients with chronic or recent transfusions due to the presence of circulating transfused RBCs and interfering allo‐ or autoantibodies (Belsito et al., 2017; Fasano & Chou, 2016). Understanding the molecular background of blood groups can lead to solving the problems associated with serological methods and can predict blood group antigens by testing DNA with a high degree of accuracy (Belsito et al., 2015; Osman et al., 2017). Molecular assays are not influenced by the presence of immunoglobulins, transfused cells, or the limitations commonly found with the antisera and can be used to determine RBC antigens even in recently transfused patients or those receiving multiple blood transfusions (Fasano & Chou, 2016; Osman et al., 2017). Therefore, using molecular blood group genotyping can support transfusion decisions and prevent alloimmunization by an antigen‐matched transfusion in these patients (Khan & Delaney, 2018; Kutner et al., 2014). Iran is a country with a considerable number of thalassemia patients, which makes it a country with the highest annual recipients of blood components. Also, there are different races in Iran (Arab, Lor, Turk, Fars, etc.), which may lead to a higher rate of antibody production because blood group frequencies may differ between races (Dorgalaleh et al., 2017; Karimi et al., 2007). Thus, the prevention of initial or additional alloantibody formation by an antigen‐matched transfusion is critical for multi‐transfused patients in Iran. Various frequencies of alloimmunization have been reported worldwide in thalassemia patients but it ranged from 4% to 50% (Makroo et al., 2016).

In the present study, we intended to determine the most clinically important antigens of Rh (D, C, c, E, and e), Kell (K, k, Kpa, and Kpb), Kidd (Jka and Jkb), and Duffy (Fya and Fyb) blood group systems by molecular assays and compare the blood group molecular genotyping with traditional serological phenotyping in alloimmunized multi‐transfused thalassemia patients who are at significant risk of making additional RBC alloantibodies.

## 2 MATERIALS AND METHODS

### 2.1 Editorial policies and ethical considerations

This study was approved by the Ethics Committee of the National Institute for Medical Research Development (NIMAD). Informed consent was taken from all patients and the demographic data were collected in case record form.

### 2.2 Patients

Two hundred alloimmunized thalassemia patients (require blood transfusion at 2–8 weeks interval) receiving treatment and follow‐up at the Tehran Adult Thalassemia Clinic, a referral center for complicated thalassemia patients, agreed to participate in this study. Peripheral blood was collected in two separate K2EDTA tubes of 5 ml. One tube was used for serological RBC phenotype and the other tube was used for DNA extraction.

### 2.3 Serological phenotyping

The Rh, Kell, Kidd, and Duffy blood group phenotypes were determined using the tube method and commercial blood group typing reagents (Immundiagnostika) according to the manufacturer's instructions. Cells with either the presence or absence of the antigens to be tested were selected from an in‐house screening cell panel that was validated with commercial panel cells (Diacell, Diamed AG). These cells were used as positive and negative controls to ensure the expected reactivity of antisera to be used in the testing. To exclude sample mix‐up all procedures for the handling and labeling of samples were revised according to the Iranian Blood Transfusion Organization (IBTO) standard operating procedure template and serological test were done for every samples twice and in discrepant cases serological tests were repeated.

### 2.4 DNA extraction

Genomic DNA was extracted from blood samples using a column‐based DNA extraction kit (YT 9040, Yekta Tajhiz Azma) and The DNA concentration of each sample was determined by the measurement of optical density at 260 and 280 nm. The extracted DNA was stored at −20°C.

### 2.5 Molecular genotyping

#### 2.5.1 Polymerase chain reaction (PCR)‐with sequence‐specific primer (PCR‐SSP)

PCR‐SSP method is based on the fact that primer extension, and consequently a successful PCR relies on a good match at the 3′ end of both primers. Therefore, only when the primers are entirely matched, the amplification of the target sequence is obtained. In this study, SSP‐PCR was performed for all 200 samples. The sequence of primers was chosen according to the previous studies and is shown in Table A1 (Ekman et al., 2002; Faas et al., 1995; Jungbauer et al., 2012). PCR was performed with 60–120 ng of DNA, 0.6 µm of the primers, and 2x Master Mix PCR (Yekta Tajhiz Azma) in a final volume of 25 µl reaction under the following conditions: 5 min at 95°C; 32 cycles of 95°C for 30 s, 60°C for 30 s, and 72°C for 30 s, and a final elongation for 5 min at 72°C. Exceptionally, the annealing temperature for reactions to detect RHE and RHe was 52°C. In each reaction, the second set of primers was included that directed the amplification of a 423 bp segment of the human growth hormone (HGH) gene as an internal positive control (Rožman et al., 2000). The amplification products were separated by electrophoresis on 0.8% agarose gel for the product of primers that detected RHD/CE and 2% agarose gel for other products. In discrepant cases, all PCR reactions were repeated to exclude sample mix‐up.

#### 2.5.2 Restriction fragment length polymorphism (RFLP)‐PCR

In order to verify the results of SSP‐PCR, RFLP‐PCR was performed for patients with incompatibility between phenotype and genotype results. PCR products amplified using primers, amplification conditions as described in the previous studies, and primer sequences are presented in Table A2 (Arnoni et al., 2013; Castilho et al., 2002; Poulter et al., 1996).

After amplification, the PCR products were digested overnight at 37°C with the appropriate restriction enzymes (Thermo Scientific), in a final volume of 32 µl using 10 µl of amplified product and enzyme in 1x buffer according to the manufacturer's instructions. The enzymes, including Pst I, Ssp I, Bsm I, Nla III, and Ban I, were employed to determine RH*D/RH*C/RH*c (insertion in RHC), RH*C/RH*c (−292G>A), KEL*01/KEL*02 (698C>T), KEL*3/KEL*4 (481C>T), and FY*A/FY*B (125G>A), respectively. Moreover, Mnl I restriction enzyme was also used to determine RH*E/RH*e (676C>G) in the RHCE gene and JK*A/JK*B (838G>A) in SLC14A1. RFLP bands were analyzed after electrophoresis in 2% agarose gel for RH*D/RH*C/RH*c, KEL*01/KEL*02, KEL*3/KEL*4, and FY*A/FY*B genotyping and in 8% polyacrylamide gel for RH*E/RH*e and JK*A/JK*B genotyping.

#### 2.5.3 DNA sequencing

To check the accuracy of protocols, 10 DNA samples, previously genotyped by PCR‐SSP and PCR‐RFLP, and all samples with undetermined phenotype and discrepancies between phenotype and genotype results were sequenced for related exons in all studied blood group systems. For KEL, Kidd, and FY sequencing, the PCR amplification was performed using primers and amplification conditions as described in the previous studies (Jalali et al., 2020; Lee et al., 1995; Nathalang et al., 2015). The Duffy silencing mutation (−67T>C) in the GATA‐1‐binding site at the promoter region of the FY gene (FY*null01) was also screened using primers, and the PCR conditions were identical to those previously described. For sequencing exons with nucleotide changes in the Rh blood group system, the RHCE gene was selected in the ensemble database (http://www.ensembl.org/index.html) and two sets of primers were designed using the Primer 3 program (http://frodo.wi.mit.edu/). Hairpin and autodimer formations were evaluated using Autodimerv1removal (http://www.cstl.nist.gov/biotech/strbase/AutoDimerHomepage/AutoDimerProgramHomepage.htm) (Table A2). For new primers, PCR was performed with 50–150 ng of DNA, 0.4 µm of the primers, and 2x Master Mix PCR (Yekta Tajhiz Azma, Iran) in a final volume of 50 µl reaction under the following conditions: 4 min at 95°C; 30 cycles of 95°C for 30 s, 67°C for 30 s, and 72°C for 30 s, and a final elongation of 8 min at 72°C. The DNA sequences were analyzed against a wild‐type sequence using the ClustalW2 multiple sequence alignment program (Ref. http://www.ebi.ac.uk/Tools/clustalw2/index.html).

## 3 RESULTS

### 3.1 Patients’ characteristics

A total of 200 alloimmunized thalassemia patients, including 81 males and 119 females with a median age of 30 ± 10.93 years (range: 4–65 years) were genotyped for Rh, Kell, Kidd, and Duffy blood group systems. Patients were diagnosed with beta‐thalassemia major (*n* = 108), beta‐thalassemia intermedia (*n* = 90), and sickle beta‐thalassemia (*n* = 2).

### 3.2 Alloantibodies

As shown in Table A3, different types of alloantibodies were identified in our patients. The majority of patients had a single alloantibody (62%), whereas 38% of them had multiple antibodies. The most common alloantibody was Anti‐K (28%), followed by Anti‐E (21%) and Anti‐D (11%).

### 3.3 Phenotype and genotype frequencies

The phenotype and genotype results of the alloimmunized thalassemia patients in this study are shown in Table 1. However, 39 samples were considered phenotypically undetermined due to mixed‐field reactions observed in Rh, Kidd, and Duffy blood group systems. Most mixed field reactions were observed in the Rh system, especially about RhCc antigens and no mixed field reaction was detected in the Kell system. All undetermined phenotypes were resolved when the patient's DNA was subjected to RBC group system genotyping. The results of phenotype and genotype frequencies in these patients are presented in Table 2. The frequency of mixed field reaction was different between thalassemia phenotypes which were 21 out of 108 (19.4%) for beta‐thalassemia major, 16 out of 90 (17.8%) for beta‐thalassemia intermedia, and 2 out of 2 (100%) for sickle beta‐thalassemia.

**TABLE 1:** Phenotyping and genotyping results on samples from 200 alloimmunized thalassemia patients

Blood group system	Alleles	antigens	Serological phenotyping	Number (percent)	Molecular genotyping	Number (percent)
Rh	RHD	D	RhD+	158 (79)	RHD+/RHCE+	160 (80)
			RhD−	42 (21)	RHD−/RHCE+	40 (20)
	RHC and RHc	C and c	C+ c−	46 (24)	RHC/RHC	58 (28.9)
			C− c+	53 (28)	RHc/RHc	52 (26.1)
			C+ c+	91 (48)	RHC/RHc	90 (45)
	RHE and RHe	E and e	E+ e−	2 (1.2)	RHE/RHE	5 (2.4)
			E− e+	158 (81.4)	RHe/RHe	151 (75.5)
			E+ e+	34 (17.4)	RHE/RHe	44 (22.2)
Kell	KEL*01 and KEL*02	K and k	K− k+	196 (98)	KEL*02/KEL*02	192 (96)
			K+ k+	3 (1.5)	KEL*01/KEL*02	8 (4)
			K− k−	1 (0.5)	–	–
	KEL*3 and KEL*4	Kpa and Kpb	Kpa− Kpb+	200 (100)	KEL*4/KEL*4	197 (98.5)
			Kpa+Kpb+	0 (0)	KEL*3/KEL*4	3 (1.5)
Kidd	JK*A and JK*B	Jka and Jkb	Jk (a+ b−)	39 (21)	JK*A/JK*A	58 (29)
			Jk (a− b+)	32 (17)	JK*B/JK*B	47 (23.5)
			Jk (a+ b+)	115 (62)	JK*A/JK*B	95 (47.5)
Duffy	FY*A and FY*B	Fya and Fyb	Fy (a+ b−)	39 (20.4)	FY*A/FY*A	47 (24)
			Fy (a− b+)	28 (14.5)	FY*B/FY*B	63 (45)
			Fy (a+ b+)	123 (64.5)	FY*A/FY*B	90 (31)
			Fy (a− b−)	1 (0.7)	—	—

**TABLE 2:** The result of phenotyping and genotyping in patients was serologically undetermined

Blood group system	Serological phenotyping	Molecular genotyping	Number (Total *N* = 39)
Rh system	C^mix^ c+	RHC/RHc	2
	C^mix^ c+	RHc/RHc	1
	C+ c^mix^	RHC/RHC	2
	C+ c^mix^	RHC/RHc	1
	C− c^mix^	RHc/RHc	2
	C^mix^ c^mix^	RHC/RHC	1
	C^mix^ c^mix^	RHc/RHc	1
	E^mix^ e+	RHe/RHe	4
	E^mix^ e+	RHE/RHe	1
	E^mix^ e^mix^	RHE/RHe	1
Kidd system	Jk (a+ b^mix^)	JK*A/JK*A	4
	Jk (a+ b^mix^)	JK*A/JK*B	3
	Jk (a^mix^ b+)	JK*A/JK*B	2
	Jk (a^mix^ b+)	JK*B/JK*B	1
	Jk (a‐ b^mix^)	JK*B/JK*B	1
	Jk (a^mix^ b^mix^)	JK*A/JK*B	2
	Jk (a^mix^ b^mix^)	JK*B/JK*B	1
Duffy system	Fy (a+ b^mix^)	FY*A/FY*A	4
	Fy (a+ b^mix^)	FY*A/FY*B	2
	Fy (a^mix^ b+)	FY*B/FY*B	2
	Fy (a^mix^ b^mix^)	FY*B/FY*B	1

### 3.4 Correlation between phenotype and genotype

Eighty‐three (41.5%) out of 200 patients showed discrepancies between genotyping and phenotyping results (Table 3). Four patients showed discrepancies in three blood group systems, 27 patients showed discrepancies in two blood group systems, and 52 patients showed discrepancies just in one system. The frequency of discrepancies was different between thalassemia phenotypes which were 58 out of 108 (53.7%) for beta‐thalassemia major, 23 out of 90 (25.6%) for beta‐thalassemia intermedia, and 2 out of 2 (100%) for sickle beta‐thalassemia. Most discrepancies were detected in Rh and Duffy blood group systems with 47 and 45 cases, respectively, and the main discrepancy was found in FY*B/FY*B allele when serologically was recognized as Fy(a+ b+). All samples with discrepancies were rechecked using the PCR‐RFLP method and DNA sequencing. No discrepancies were found between the three genotyping methods. All discrepant samples in the Duffy system were also evaluated for silencing mutation (−67T>C) in the GATA‐1‐binding motif at the promoter region of the FY gene but no positive cases were detected.

**TABLE 3:** The result of phenotyping and genotyping in patients with discrepancies

Blood group system	Serological phenotyping	Molecular genotyping	Number (Total *N* = 132)
Rh system	RhD+^a^	RHD−	1
	RhD−	RHD+	2
	C+ c+	RHC/RHC	9
	C+ c+	RHc/RHc	7
	C− c+	RHC/RHc	7
	C− c+	RHC/RHC	2
	C+ c−	RHC/RHc	1
	C+ c−	RHc/RHc	1
	E− e+	RHE/RHE	10
	E+ e+^b^	RHe/RHe	7
	E+ e−	RHe/RHe	1
Kell system	K− k+	KEL*01/KEL*02	6
	K− k−	KEL*02/KEL*02	1
	Kpa− Kpb+	KEL*3/KEL*4	3
Kidd system	Jk (a+ b+)	JK*A/JK*A	15
	Jk (a+ b+)	JK*B/JK*B	12
	Jk (a+ b−)	JK*A/JK*B	1
	Jk (a− b+)	JK*A/JK*B	1
Duffy system	Fy (a+ b+)	FY*B/FY*B	27
	Fy (a+ b+)	FY*A/FY*A	6
	Fy (a+ b−)	FY*A/FY*B	7
	Fy (a+ b−)	FY*B/FY*B	1
	Fy (a −b+)	FY*A/FY*B	2
	Fy (a+ b^mix^)	FY*B/FY*B	2

^a^ Patients with the RhD+ RHD− discrepancy had anti‐D.

^b^ 3 of 7 patients with the E+ e+ RHe/RHe discrepancy had anti‐E.

In 101 cases, the discrepancies had clinical significance meaning that these patients had the potential of alloimmunization and in four cases, the discrepancies resulted in antibody production. Three patients phenotyped as E+ e+ and genotyped as RHe/RHe had anti‐E, and one patient serologically phenotyped as RhD‐positive and genotyped as RHD‐negative, had anti‐D in his serum.

## 4 DISCUSSION

This study indicated the relevance of performing molecular analysis to determine the most clinically important blood group systems, including Rh (D, C, c, E, and e), Kell (K, k, Kpa, and Kpb), Kidd (Jka and Jkb), and Duffy (Fya and Fyb) in Iranian alloimmunized transfusion‐dependent thalassemia patients. We observed discrepancies between phenotyping and genotyping results in a total of 132 alleles in 83 (41.5%) patients mainly in Rh and Duffy systems with 47 and 45 cases, respectively; however, there was a complete agreement between the results of SSP‐PCR, RFLP‐PCR, and DNA sequencing. In 101 of these discrepancies, the patients were at risk of making antibodies and in four cases, the discrepancies resulted in antibody production. All undetermined serologic phenotypes because of the mixed field reactions were resolved by genotyping.

RBC alloantibody formation is a major complication associated with repeated transfusions in multi‐transfused thalassemia patients and limits the availability as well as the safety of subsequent transfusions (Kutner et al., 2014; Osman et al., 2017). In these patients, the seriousness of the alloimmunization problem has led to recommendations that patients be transfused with the blood of donors whose RBC antigens are more closely matched to those of the recipients (Guelsin et al., 2010). In this regard, extended red cell typing to transfuse phenotypically matched blood has been performed in chronically transfused patients (O'suoji et al., 2013). However, several studies have demonstrated that the majority of blood banks do not routinely perform extended typing of RBCs (Patel et al., 2016). Limitations that are cited include the cost of extended antigen matching compared with standard red cell typing and the longer time needed to perform the procedures (Castro et al., 2002). Among all the blood group systems, Rh, Kell, Kidd, and Duffy are the clinically significant groups that contain the antigens to provoke the most severe transfusion reactions (Ye et al., 2016).

In our study, all patients had a history of alloimmunization, and 87% of antibodies were formed against Rh, Kell, Kidd, and Duffy blood group system antigens. The most common antibodies in our study were anti‐K and anti‐E, which have previously been reported in the literature for individuals with thalassemia (Davoudi‐Kiakalayeh et al., 2017; Schonewille, 2008; Spanos et al., 1990; Thompson et al., 2011). Therefore, the selection of these most clinically important antigens for extended red cell typing is more cost‐effective and can significantly reduce alloimmunization.

Many previous studies have demonstrated that accurate phenotyping with the classical hemagglutination method is not reliable in multi‐transfused patients due to the presence of donor‐derived erythrocytes in the patient's circulation, interfering allo‐ or autoantibodies, and the lack of specific antisera (Bakanay et al., 2013; Fasano & Chou, 2016).

In this study, we indicated the relevance of performing molecular analysis to determine the most clinically important blood group systems, including Rh (D, C, c, E, and e), Kell (K, k, Kpa, and Kpb), Kidd (Jka and Jkb), and Duffy (Fya and Fyb) in Iranian alloimmunized transfusion‐dependent thalassemia patients.

In the present study, we observed discrepancies between phenotyping and genotyping results in a total of 132 alleles in 83 (41.5%) patients; however, there was a complete agreement between the results of SSP‐PCR, RFLP‐PCR, and DNA sequencing. Previous studies have shown that, in multi‐transfused patients, the rate of discrepancies between phenotyping and genotyping results were variable and reported at 42.1%–90% (Bakanay et al., 2013; Castilho et al., 2002; Guelsin et al., 2010; Kulkarni et al., 2018). In this study, the blood group phenotype was considered undetermined in 39 cases using the serological methods because of the mixed field reaction; however, all undetermined phenotypes were resolved by molecular genotyping.

It should always be taken into account that in the healthy population, there is a high correlation between the molecular genotyping and serology (99.95%) and the discrepancies usually were observed in the transfusion‐dependent patients owing to the recent or chronic transfusion, as well as the presence of a mixed population of the erythrocytes (Belsito et al., 2017).

Forty‐seven of the discrepancies occurred in the Rh system, and most of them (10 cases) were related to the state of the phenotype E− e+ and genotype RHE/RHE; it seems that the phenotype reactivity was due to donor RBCs in the circulation of the patients because of the phenotype‐matched transfusion. However, the segments of the transfused units were not available to confirm it. Nonetheless, using molecular typing and accurate determination of E (RHE) antigen, we can remove the previous obligation to search for E‐negative RBC units in these patients. This discrepancy has been seen previously in the study conducted by Bakanay et al., (2013) in two patients. Two patients who had been previously phenotyped as RhD‐negative were genotyped as RHD+ in the study. According to several studies, in addition to the presence of transfused RBCs, this state can be also observed in the patients carrying week D or partial D variants; thus, in these patients, further assessments are needed to identify the accurate phenotype (Bakanay et al., 2013; Guelsin et al., 2010).

Ten cases were found to have discrepancies in the Kell blood group system that most of them (six cases) were related to the state of the K− k+ phenotype and KEL*01/KEL*02 genotype (Reyhaneh et al., 2020). Consequently, using molecular typing and accurate determination of K (KEL*01) antigen, searching for K‐negative RBC units in these patients, are not needed and saving a significant number of K‐negative RBC units. Bakanay et al., (2013), Belsito et al., (2015), and Guelsin et al., (2010) have also found the same discrepancy in their studies.

Three patients who had been phenotyped as Kpa− Kpb+ were genotyped as KEL*03/KEL*04. According to this finding, in our study, serological typing could not detect Kpa (KEL*03) antigens in any patients. Kpa is a low‐incidence RBC antigen that affects all Kell antigens encoded by the same haplotype and can markedly weaken their expression (Körmöczi et al., 2009; Sunassee et al., 2018). Accordingly, in some cases, it can cause a serological mistyping. Thus, it is important to detect this antigen accurately. According to our results and those reported by Günther et al., in the patients with Kpa (KEL*03) antigen, very sensitive serological methods or molecular genetic techniques may be required for reliable Kell typing (Körmöczi et al., 2009).

Twenty‐nine discrepancies occurred in the Kidd blood group system and most of them (15 cases) were related to the state of the phenotype Jk (a+ b+) when genotyped as JK*A/JK*A. Twelve discrepant cases were also phenotyped as Jk (a+ b+) and genotyped as JK*B/JK*B. Kidd antigens have been frequently implicated in delayed hemolytic transfusion reactions (DHTR) (Pineda et al., 1999; Vucelic et al., 2005). These antibodies fall rapidly to undetectable levels in the plasma following to immunization (Vucelic et al., 2005). Therefore, accurate determination of Kidd antigens by molecular methods is crucial to prevent further transfusion reactions.

After the Rh system, most of the discrepancies occurred in the Duffy blood group system (45 cases), of which most of them (27 cases) were related to the state of Fy (a+ b+) phenotype and FY*B/FY*B genotype. The same discrepancy was also seen in the study conducted by Guelsin et al., (2010). The high failure rate of serological typing has been reported for the Duffy blood group system in multiply transfused patients (Castilho et al., 2002; Osman et al., 2017). Therefore, using molecular methods for accurate Duffy typing is of great importance. The Duffy silencing mutation (−67T>C) in the GATA‐1‐binding motif at the promoter region of the FY gene (FY*null01) inhibits the expression of Duffy glycoprotein on the erythrocytes while preserving the expression of both Fya and Fyb in other tissues (Bakanay et al., 2013; Belsito et al., 2015). In the presence of a normal GATA‐1‐binding motif, phenotypes and genotypes agree, but when the GATA‐1 motif is mutated a pseudo‐discrepancy is observed due to the absence of the FY gene expression in the erythroid lineage (Castilho et al., 2000). However, patients carrying such phenotypes do not need blood units of negative phenotype because of the expression of Duffy antigens in other body tissues (Castilho et al., 2000). Accordingly, the availability of blood units for such patients was facilitated. In the present study, we evaluated all discrepant samples in the Duffy system for this mutation but no positive cases were detected.

In patients who had been transfused with genotype mismatched RBC units, because of the observed genotype‐phenotype discrepancies, 101 patients had the potential to induce alloantibodies. Surprisingly, just in four patients (anti‐E in 3 patients and anti‐D in 1 patient), it results in antibody production because many factors affect the risk of alloimmunization, including antigen immunogenicity, the duration of transfusion therapy, and genetic and environmental factors (Osman et al., 2017).

It should be considered that genotyping analysis can only predict the blood group phenotype and in rare situations, genotype determination may not be correlated with antigen expression in the RBCs (Casas et al., 2015; Reid & Denomme, 2011). Moreover, in the majority of DNA‐based assays, specific nucleotide(s) or genomic sites are considered (Reid & Denomme, 2011). Therefore, incorrect prediction of the phenotype and detection of an apparent grossly normal allele is always possible, which can be silenced by mutations beyond the primer binding sites (Jungbauer et al., 2012). In this study, the sequencing of the entire gene was not carried out. Therefore, it is possible that discrepancies between the phenotype and genotype results are made by silent variants that are neglected for detection.

In this study, one patient was found with the K−k− in phenotype and the KEL*01/KEL*02 in genotype, and another patient with the Fy (a− b−) in phenotype and the FY*B/FY*B in genotype with normal GATA‐1‐binding motif; thus, in these patients, further assessments are needed for the accurate determination of antigen expression status considering the potential silent variants.

## 5 CONCLUSION

According to the obtained results, using DNA‐based antigen testing is effective to improve the effectiveness and reliability of blood group antigen typing and resolve undetermined blood group phenotypes in transfusion‐dependent cases. Although genotyping is associated with several advantages, it cannot be regarded as an alternative; nonetheless, it is applicable as a complementary tool to overcome the limitations of serological assays. Also, using blood group genotyping combined with serological assays in transfusion‐dependent patients can result in antigen‐matched RBC transfusions and can improve patients care.

## CONFLICT OF INTEREST

The authors report no conflicts of interest in this work.

## AUTHOR CONTRIBUTIONS

SR: methodology, validation, writing—review & editing. OA: conceptualization, project administration. DTR, JSF, MF, GS: methodology, validation. AA: methodology. NA: conceptualization, supervision.

## Data Availability

Data are available on request from the authors.

## APPENDIX A

**TABLE A1:** PCR primers used in SSP‐PCR for Rh, Kell, Kidd, and Duffy blood group genotyping

Blood group system	Antigen	Allele	Nucleotide change	Forward/Reverse	Primer sequence	Product size (bp)
Rh	RhD	RHD/CE(intron4)	Deletion in RHD	F	5'‐ACGATACCCAGTTTGTCT‐3’	D: 600 CE: 1200
				R	5'‐TGACCCTGAGATGGCTGT‐3’	
	RhC	RHD/CE (intron2)	Insertion in RHC	F	5'‐TCAGGGGAGGGGCGTATCTTATTC‐3’	C: 594 D or c: 485
				R	5'‐GAACATGCCACTTCACTCCAG‐3’	
		RHC	48C (G)	F	5'‐GCGCTGCCTGCCCCTCTTC‐3’	114
				R	5'‐TAGGATGCCACGAGCCCCTTT‐3’	
	Rhc	RHc	48C>G	F	5'‐TGTGATGACCACCTTCCCTGG‐3’	179
				R	5'‐TAGGCCAAGATCTGACCG‐3’	
	RHE	RHE	676C (G)	F	5'‐CCAAGTGTCAACTCTC‐3’	108
				R	5'‐TGACCCTGAGATGGCTGT‐3’	
	Rhe	RHe	676C>G	F	5'‐CCAAGTGTCAACTCTG‐3’	141
				R	5'‐CATGCTGATCTTCCT‐3’	
Kell	K	KEL*01	578C>T	F	5^'^‐ACTCATCAGAAGTCTCAGCA‐3^’^	360
				F	5^'^‐CTAGAGGGTGGGTCTTCTTCC‐3^’^	
	k	KEL*02	578C (T)	R	5^’^‐CTCATCAGAAGTCTCAGCG‐3^’^	360
				F	5^'^‐CCAAGGCCAAGTGTCAGTGC‐3^’^	
	Kpa	KEL*3	841C>T	R	5^'^‐ TGTCAATCTCCATCACTTCAT‐3^’^	620
				F	5^'^‐CTGCCCGCACAGGTGGC‐3^’^	
	Kpb	KEL*4	841C (T)	R	5^'^‐CAATCTCCATCACTTCACG‐3^′^	620
				F	CTGCCCGCACAGGTGGC‐3^′^‐5^′^	
Kidd	Jka	JK*A	838G (A)	F	GTCTTTCAGCCCCATTTGCGG‐3^′^‐5′	528
				R	CCAAGGCCAAGTGTCAGTGC‐3^′^‐5′	
	Jkb	JK*B	838G>A	F	AGTCTTTCAGCCCCATTTGCGA‐3′‐5′	528
				R	CCAAGGCCAAGTGTCAGTGC‐3′‐5′	
Duffy	Fya	FY*A	125G (A)	F	5′‐CTCATTAGTCCTTGGCTCTTAT‐3′	713
				R	5′‐CAGCTGCTTCCAGGTTGGGAC ‐3′	
	Fyb	FY*B	125G>A	F	5′‐TCATTAGTCCTTGGCTCTTAT‐3′	713
				F	5′‐CAGCTGCTTCCAGGTTGGgAT‐3′	
—	—	Control (Hgh)	—	R	5′‐TGCCTTCCCAACCATTCCCTTA‐3′	434
				F	5′‐CCACTCACGGATTTCTGTTGTGTTTC‐3′	

**TABLE A2:** PCR primers used in RFLP‐PCR and DNA sequencing for Rh, Kell, Kidd, and Duffy blood group genotyping

Method	Alleles	Nucleotide	Forward/reverse	Primer sequence	Product size (bp)
RFLP‐PCR	RHD/RHC/RHc	Insertion in RHC	F	5′‐GTGCCACTTGACTTGGGACT‐3′	C: 1174 D or c: 1068
			R	5′‐GTGGACCCAATGCCTCTG‐3′	
	RHC/RHc	−292G>A	F	5′‐CTGTGTAACTATGAGGAGTCAA‐3′	302
			R	5′‐AGAGGGCATTCTATTCCTTTGA‐3′	
	RHE/RHe	676C>G	F	5′‐GGCAACAGAGCAAGAGTCCA‐3′	474
			R	5′‐CTGATCTTCCTTTGGGGGTG‐3′	
	KEL*01/KEL*02	578C>T	F	5′‐AAGCTTGGAGGCTGGCGCAT‐3′	156
			R	5′‐CCTCACCTGGATGACTGGTG‐3′	
	KEL*3/KEL*4	841C>T	F	5′‐AGGAGAAAAGCAGGGACCTC‐3′	364
			R	5′‐AGGGGATGGAGTCAGAGACA‐3′	
	JK*A/JK*B	838G>A	F	5′‐ TGAGATCTTGGCTTCCTAGG ‐3′	210
			R	5′‐ ATTGCAATGCAGGCCAGAGA ‐3′	
	FY*A/FY*B	125G>A	F	5′‐TCCCCCTCAACTGAGAACTC ‐3′	392
			R	5′‐AAGGCTGAGCCATACCAGAC ‐3′	
DNA sequencing	RHC/RHc (exon1)	48C>G	F	5′‐TCCATAGACAGGCCAGCACAGC‐3′	340
			R	5′‐GCTATTTGCTCCTGTGACCACTGT‐3′	
	RHE/RHe (exon5)	676C>G	F	5′‐TTCTGGCCAACCACCCTCTCTG‐3′	367
			R	5′‐CCTGTGACCACCCAGCATCTTCC‐3′	
	KEL*01/KEL*02 (exon6)	578C>T	F	5′‐TTTAGTCCTCACTCCCATGCTTCC‐3′	740
			R	5′‐TATCACACAGGTGTCCTCTCTTCC‐3′	
	KEL*3/KEL*4 (exon8)	841C>T	F	5′‐ATATTCCCCACCTCCCCACACCTG‐3′	800
			R	5′‐ATCTACGGTGCTCAGGCTCTCCTC‐3′	
	JK*A/JK*B (exon9)	838G>A	F	5′‐TTAGTCCTGAGTTCTGACCCCT‐3′	218
			R	5′‐GATCCTGTAGTCATGAGCAGC‐3′	
	FY*A/FY*B (exon2)	125G>A	F	5′‐ GTGTAGTCCCAACCAGCCAA‐3′	931
			R	5′‐AGGATACCCAGGACACTGGT‐3′	
	FY*null01 (promoter)	−67T>C	F	5′‐ GTGTAGTCCCAACCAGCCAA ‐3′	264
			R	5′‐ GCC CCATACTCACCCTGTG ‐3′	

**TABLE A3:** Antibody specification and frequency in 200 alloimmunized thalassemia patients in this study

Antibody specificity	Frequency	
	Number	Percent
Anti‐K	65	28%
Anti‐E	53	21%
Anti‐D	25	11%
Anti‐Kp^a^	17	7%
Anti‐C	17	7%
Anti‐c	11	5%
Anti‐JK^a^	10	4%
Anti‐S	10	4%
Anti‐C^w^	6	2%
Anti‐JK^b^	6	2%
Anti‐ e, Anti‐Fyb, Anti‐s	2	1%
Nonspecific clinically significant antibody	15	6%
Total	237	100%
